# Single-Session Percutaneous Mechanical Thrombectomy for Acute and Subacute Deep Vein Thrombosis: Clinical Outcomes and Predictive Factors of Recurrence

**DOI:** 10.5334/jbsr.3213

**Published:** 2023-08-16

**Authors:** Sangjoon Lee, Youngjong Cho, Hyoung Nam Lee, Sung-Joon Park, Hwan Hoon Chung, Hyerim Park

**Affiliations:** 1 Vascular Center, The Eutteum Orthopedic Surgery Hospital, Paju-si, Republic of Korea; 2 Department of Radiology, University of Ulsan College of Medicine, Gangneung Asan Hospital, Gangneung-si, Republic of Korea; 3 Department of Radiology, Soonchunhyang University College of Medicine, Cheonan Hospital, Cheonan-si, Republic of Korea; 4 Department of Radiology, Korea University College of Medicine, Korea University Ansan Hospital, Ansan-si, Republic of Korea

**Keywords:** Endovascular procedures, venous thrombosis, thrombectomy, treatment outcome, risk factors

## Abstract

**Objectives:**

To evaluate the efficacy and safety of single-session percutaneous mechanical thrombectomy (PMT) for deep vein thrombosis (DVT), to compare clinical outcomes and recurrences between acute and subacute DVT, and to identify factors predicting recurrence.

**Materials and Methods:**

From January 2018 to March 2021, 100 consecutive patients (age: 64.64 ± 17.28 years; male, 42%) with symptomatic DVT who underwent single-session PMT were enrolled for this study. These patients were divided into an acute DVT group (< 14 days, *n* = 75) and a subacute DVT group (15–28 days, *n* = 25).

**Results:**

A large-bore aspiration thrombectomy was used in 80 (80%) cases, Angiojet (Boston Scientific, Marlborough, MA, USA) device in one (1%) case, and a combination of both techniques in 19 (19%) cases. The anatomic success rate was 97% and the clinical success rate was 95%. There were no major complications. Clinical outcomes were not different between the two groups. The recurrence-free survival rate in the acute DVT group was significantly (*p* = 0.015) better than that in the subacute DVT group. The anatomic success (HR, 52.3; 95% CI, 3.82–715.21; *p* = 0.003) and symptom duration (HR, 17.58; 95% CI, 1.89–163.34; *p* = 0.012) were predictive factors associated with recurrence.

**Conclusions:**

Single-session PMT is safe and effective for immediate symptom relief in acute and subacute DVT patients. However, recurrence occurred more frequently in patients with subacute DVT than in those with acute DVT. Anatomic success of the procedure and duration of symptoms were independent predictors of DVT recurrence.

## Introduction

Endovascular treatment has been one of the major therapeutic methods for deep vein thrombosis (DVT), employing techniques like catheter-directed thrombolysis (CDT) and percutaneous mechanical thrombectomy (PMT) using large-bore catheters or thrombectomy devices. CDT can provide short-term symptom relief as well as improvement in quality of life, especially for iliofemoral DVT [1]. However, bleeding risk is higher in patients treated with CDT than in those treated with anticoagulants alone. Reported rate of major bleeding complications ranges from 1.7% to 5.2% [1,2,3]. Single-session PMT can be an effective option since it allows the use of a minimal dose of drugs, thereby reducing potential bleeding complications.

The most optimal indications for endovascular treatment include acute iliofemoral DVT in ambulatory patients with low bleeding risk and long-life expectancy [4]. Patients with highly symptomatic subacute DVT may also benefit from endovascular treatment [5]. Several previous studies have reported that even in subacute patients, a significant portion of thrombus could be successfully removed through PMT [6,7]. However, clinical outcomes and recurrences after PMT for acute and subacute DVT have been rarely compared.

The purpose of this study was to evaluate the efficacy and safety of single-session PMT for DVT, to compare clinical outcomes and recurrences between acute and subacute DVT, and to identify factors predicting recurrence.

## Materials and Methods

### Study participants

Inclusion criteria were: 1) patients who underwent a single-session PMT between January 2018 and March 2021 and 2) those with a diagnosis of DVT on ultrasound or CT examination. Symptoms included swelling and/or tenderness of lower extremities. During the study period, a single-session PMT performed for patients with DVT who have symptoms lasting less than 28 days. However, patients with isolated distal DVT, poor functional status, and a life expectancy less than one year were not considered suitable candidates. From three hospitals, 105 consecutive patients were collected. Five patients were excluded (four patients were lost to follow-up after discharge and one patient died due to underlying disease during index hospitalization). Finally, a total of 100 patients (age: 64.64 ± 17.28 years; male, 42%) were enrolled and divided into an acute (< 14 days) DVT group (*n* = 75) and a subacute (15–28 days) DVT group (*n* = 25) [6].

### Treatment strategies

All patients received initial and long-term anticoagulation therapy with vitamin K antagonists, oral anticoagulants, or low-molecular-weight heparin. Prior to the procedure, 2000–3000 IU of heparin (Clexane; Sanofi-Aventis, Paris, France) was administered intravenously. Inferior vena cava (IVC) filters were inserted in patients who had a limited cardiopulmonary reserve or had undergone standalone PMT without thrombolytic agent [8]. Under local anesthesia, the popliteal vein was accessed in the prone position. The femoral or jugular veins were accessed in the supine position if the prone position was not possible.

**Figure 1:**
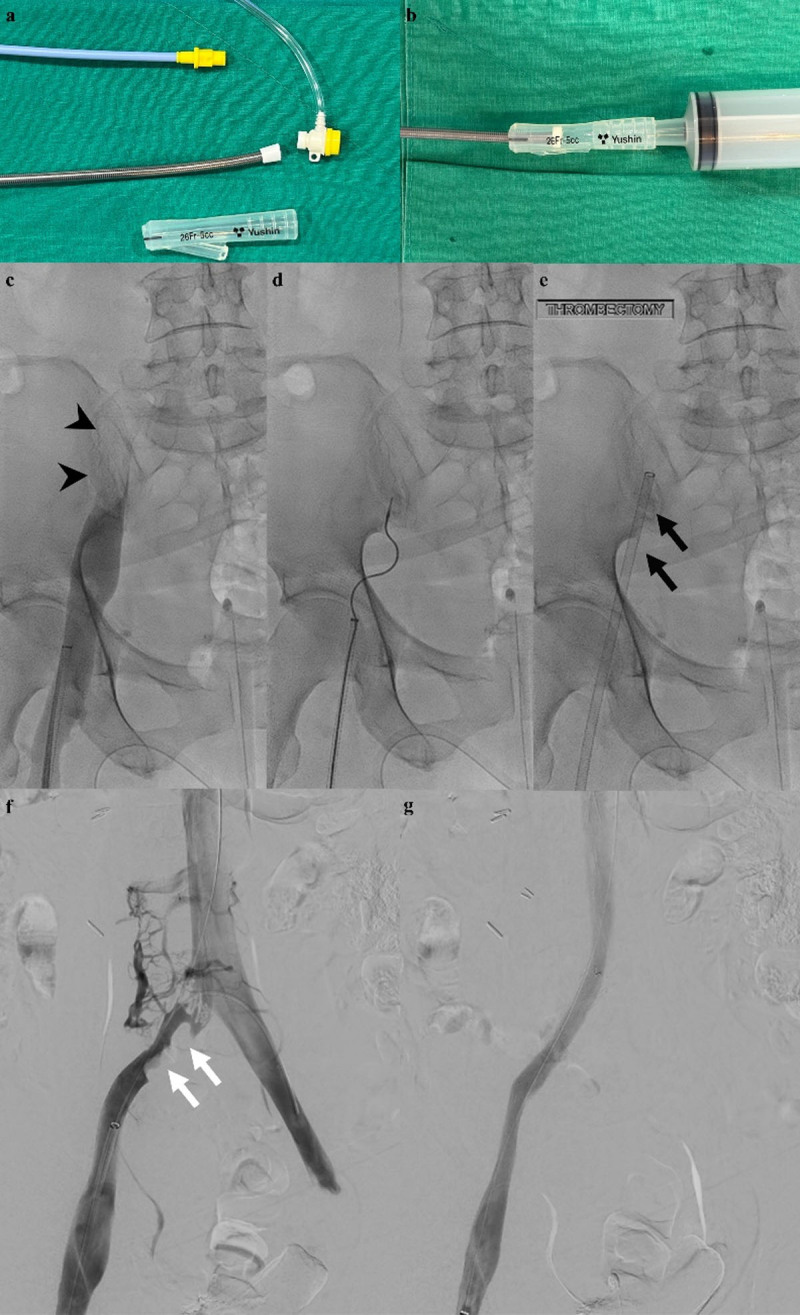
A 71-year-old woman with subacute deep vein thrombosis. **(a, b)** Modification of 11-F catheter using a hub from the 24-F Foley catheter and a 50-cm^3^ enema syringe. **(c, d)** Maceration of thrombus with a rotational thrombectomy device. **(e)** Aspiration thrombectomy with a 11-F introducer catheter. **(f, g)** Placement of a 12 mm bare metal stent at right common iliac vein stenosis.

The following methods were used alone or in combination: 1) large-bore aspiration thrombectomy; and 2) Angiojet thrombectomy system (Boston Scientific, Marlborough, MA, USA). For large-bore aspiration thrombectomy, an 11F introducer sheath (Super Arrow-Flex percutaneous introducer set; Arrow International, Reading, MA, USA) was used (Figure 1) [6,9]. At the discretion of the attending physician, 5 mg of recombinant tissue plasminogen activator (Actilyse, Boehringer Ingelheim, Ingelheim, Germany) or 100,000–150,000 IU of urokinase (GC Biopharma Corp., Yongin, Korea) was administered directly into the thrombus through an infusion catheter (Multi-Sideport; Cook Medical, Bloomington, IN, USA) or the Angiojet device.

Maceration of the residual thrombus was performed as needed using a pigtail angiographic catheter (Cook Medical, Bloomington, IN, USA), an Omni flush catheter (AngioDynamics, Queensbury, New York), a balloon catheter (Mustang; Boston Scientific, Marlborough, MA, USA), Cleaner 15 (Argon medical devices, TX, USA) and/or a Trerotola device (Arrow International, Reading, Pennsylvania). Bare metal stents with a diameter of 12 to 14 mm were implanted in cases where there was significant residual stenosis in iliac veins after angioplasty.

### Definitions

The lower extremity thrombosis (LET) classification was used to classify the level of thrombosis (class I, calf vein thrombosis; class II, popliteal and femoral vein thrombosis; class III, common femoral/iliac vein obstruction; class IV, IVC thrombosis) [10]. Laterality refers to the side of the body affected (right, left, or bilateral). For bilateral DVT, the leg with the most cranial involvement was considered the index leg. Adjunctive procedure refers to balloon angioplasty and/or subsequent stent placement for iliac vein stenosis (≥ 50% on venography).

Anatomic success was defined as more than 50% removal of the thrombus and restoration of iliofemoral venous flow. Clinical success was defined as resolution of the symptom at discharge. Complications were classified as minor or major ones according to the guidelines of Society of Interventional Radiology [11]. The length of post-procedure hospital stay was defined as the number of days from PMT to hospital discharge. Upon follow-up imaging after discharge, recurrence was defined as thrombosis of a new venous segment or a previously involved segment that showed symptomatic and imaging improvement [5].

### Statistical analysis

Comparative analyses were performed using two-sample t-test or Mann-Whitney U test for continuous variables, and chi-square test or Fisher’s exact test for categorical variables. Recurrence-free survival rates were analyzed using Kaplan-Meier survival analysis. Cox proportional hazards regression analyses with forward stepwise selection were performed to identify predictive factors for recurrence. The variance inflation factor was calculated to test the multicollinearity. A *p*-value < 0.05 was considered statistically significant. All statistical analyses were executed using R version 3.6.3 software (Foundation for Statistical Computing).

## Results

Baseline characteristics are presented in Table 1. Eleven (11%) patients were absolutely or relatively contraindicated to thrombolysis because of active internal bleeding (hemoptysis), recent intracranial surgery, intracranial hemorrhage, or intracranial abscess. Details of procedures are summarized in Table 2. The IVC filter was inserted in 91 (91%) cases prior to the procedure. A large-bore aspiration thrombectomy was used in 80 (80%) cases, Angiojet device was used in one (1%) case. A combination of both techniques was used in 19 (19%) cases.

**Table 1:** Baseline characteristics of study population.


PARAMETER	ALL	ACUTE DVT	SUBACUTE DVT	*p*-VALUE

	(*n* = 100)	(*n* = 75)	(*n* = 25)	

**Age, years**	64.64 ± 17.28	66.61 ± 16.89	58.72 ± 17.39	0.045*

**Gender (female)**	58 (58%)	48 (64%)	10 (40%)	0.061

**BMI, kg/m2**	25.31 ± 4.36	24.79 ± 3.52	26.89 ± 6.06	0.111

**Provoked**	53 (53%)	38 (50.7%)	15 (60%)	0.563

**First episode**	95 (95%)	71 (94.7%)	24 (96%)	>0.99

**Lateralization**				0.818

Left	90 (90%)	66 (88%)	24 (96%)	

Right	8 (8%)	7 (9.3%)	1 (4%)	

Bilateral	2 (2%)	2 (2.7%)	0 (0%)	

**LET classification**				0.291

Class II	13 (13%)	8 (10.7%)	5 (20%)	

Class III	66 (66%)	49 (65.3%)	17 (68%)	

Class IV	21 (21%)	18 (24%)	3 (12%)	

**CIx for thrombolytic therapy**				0.861

Relative	7 (7%)	6 (8%)	1 (4%)	

Absolute	4 (4%)	3 (4%)	1 (4%)	


DVT = deep vein thrombosis; BMI = body mass index; LET = lower extremity thrombosis; CIx = contraindications. * *p* < 0.05 means statistical significance.

**Table 2:** Details of procedure.


PARAMETER	ALL	ACUTE DVT	SUBACUTE DVT	*p*-VALUE

	(*n* = 100)	(*n* = 75)	(*n* = 25)	

**IVC filter**	91 (91%)	70 (93.3%)	21 (84%)	0.222

**Thrombolytic agent**	16 (16%)	10 (13.3%)	6 (24%)	0.22

**Maceration**	40 (40%)	30 (40%)	10 (40%)	>0.99

**Iliac procedure**	64 (64%)	50 (66.7%)	14 (56%)	0.471

**Thrombectomy technique**				0.831

Large-bore aspiration	80 (80%)	59 (78.7%)	21 (84%)	

Angiojet device	1 (1%)	1 (1.3%)	0 (0%)	

Combined	19 (19%)	15 (20%)	4 (16%)	


DVT = deep vein thrombosis; IVC = inferior vena cava.

Procedure outcomes are summarized in Table 3. Anatomic success was achieved in 97 (97%) cases. There were three cases of anatomic failures in which hydrophilic guidewires could not pass through obstructed segments. Clinical success was achieved in 95 (95%) cases. Mean post-procedure hospital stay was 4.12 ± 3.29 days. In 58% of cases, patients were discharged within three days. One minor complication resulted from an inadvertent guidewire penetration, causing contrast extravasation from a small branch of femoral vein. Repeat venography confirmed spontaneous hemostasis. Comparative analysis showed no significant differences in procedure outcomes between the two groups.

**Table 3:** Clinical outcomes after single-session percutaneous mechanical thrombectomy.


PARAMETER	ALL	ACUTE DVT	SUBACUTE DVT	*p*-VALUE

	(*n* = 100)	(*n* = 75)	(*n* = 25)	

**Anatomical success**	97 (97%)	73 (97.3%)	24 (96%)	>0.99

**Clinical success**	95 (95%)	73 (97.3%)	22 (88%)	0.098

**Complication**				>0.99

Major	0 (0%)	0 (0%)	0 (0%)	

Minor	1 (1%)	1 (1.3%)	0 (0%)	

**Hospital stays, days**	4.12 ± 3.29	3.84 ± 2.57	4.96 ± 4.83	0.716


DVT = deep vein thrombosis.

**Figure 2:**
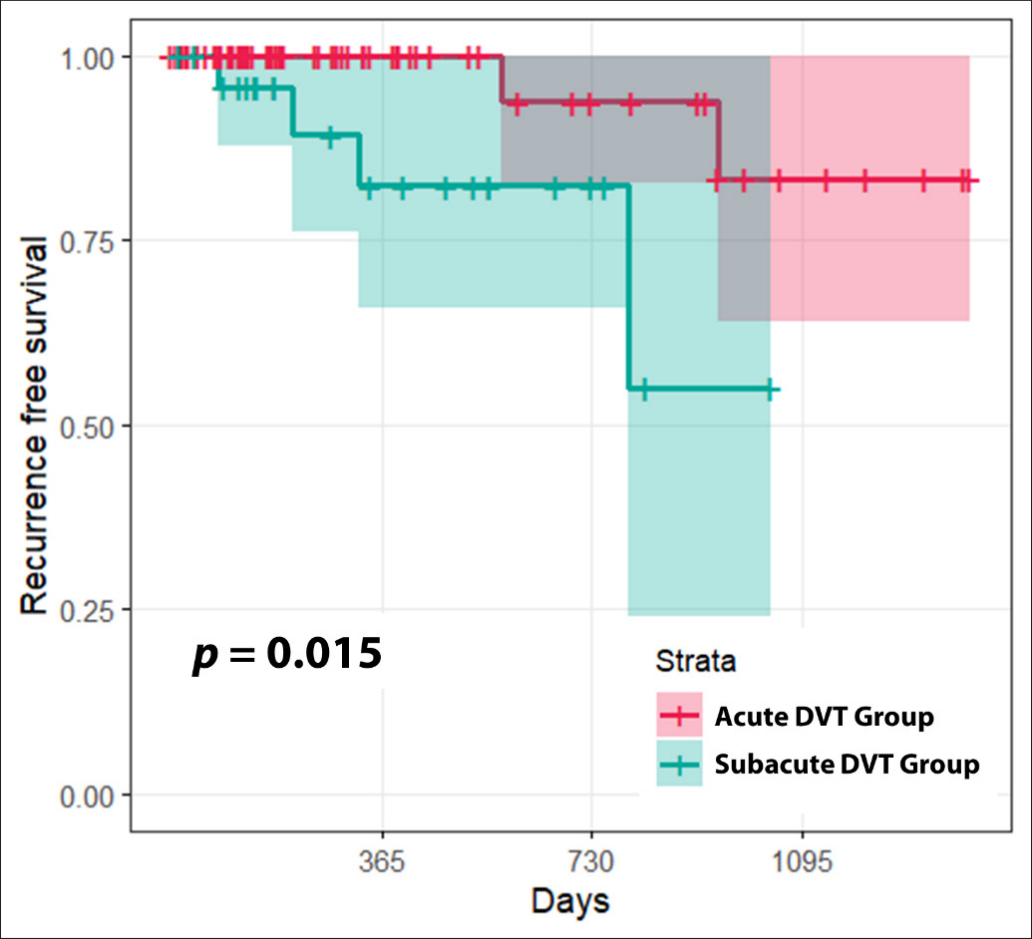
Kaplan-Meier curves showing recurrence-free survival rates.

The Kaplan–Meier survival curve stratified based on the symptom duration is shown in Figure 2. The median follow-up period was 6.8 months (IQR: 3.5–17.6 months). Recurrent DVT was observed in six (6%) patients. The recurrence-free survival rate in the acute DVT group was significantly better than that in the subacute DVT group (*p* = 0.015). Results of Cox proportional hazards regression models are shown in Table 4. Anatomic success (HR, 52.3; 95% CI, 3.82– 715.21; *p* = 0.003) and symptom duration (HR, 17.58; 95% CI, 1.89– 163.34; *p* = 0.012) were predictive factors associated with recurrence. There was no multicollinearity.

**Table 4:** Predictive factors for recurrence after single-session percutaneous mechanical thrombectomy.


PARAMETER	UNIVARIABLE ANALYSIS			MULTIVARIABLE ANALYSIS		
	
	HR	95% CI	*p*-VALUE	HR	95% CI	*p*-VALUE

**Age**	0.939	0.891–0.99	0.02*			

**Gender (female)**	0.906	0.181–4.541	0.904			

**BMI**	1.01	0.818–1.247	0.924	0.831	0.655–1.053	0.126

**Sx. duration (subacute)**	6.755	1.174–38.851	0.032*	17.58	1.892–163.34	0.012*

**Etiology (unprovoked)**	0.622	0.114–3.41	0.584			

**Lateralization (left)**	6.337	0.656–61.269	0.111			

**LET classification (IV)**	2.675	0.536–13.363	0.231			

**Iliac procedure**	0.526	0.059–4.68	0.565			

**Anatomic success (failure)**	9.729	1.706–55.481	0.01*	52.298	3.824–715.21	0.003*


BMI = body mass index; Sx. = symptom; LET = lower extremity thrombosis. * *p* < 0.05 means statistical significance.

## Discussion

Single-session PMT can remove thrombus with use of limited or no thrombolytic drug, which may be especially useful for patients at a high bleeding risk. In this study, iliofemoral venous flow was restored by removing thrombus through single-session PMT in 97% of patients. Ninety-five percent of patients experienced immediate symptom relief over the course of hospital stay of 4.12 ± 3.29 days. After the procedure, 58% of patients were discharged within three days. Accordingly, single-session PMT seems to be highly effective in terms of immediate symptom relief, leading to early discharge.

Serious adverse events such as major bleeding episodes and additional pulmonary embolisms were not observed in this study. Thrombolysis was contraindicated in 11% of study subjects. Single-session PMT with use of limited or no thrombolytic agent appears to be quite useful in this specific subgroup of patients. Results of the present study indicate that concomitant use of minimal thrombolytic agents or IVC filters may reduce the risk of pulmonary embolism. Previous studies of single-session PMT have also found that major adverse events are very rare [12,13,14]. A recent study with an Angiojet device has found that the incidence of major bleeding or pulmonary embolism is less than 2% [14].

Anatomic and success rates of acute and subacute DVT patients were comparable in this study. Since the thrombus adheres and organizes after the acute stage, treatment of non-acute DVT is challenging. Rather than CDT alone, other endovascular techniques should be considered for non-acute DVT, which may explain the acceptable outcomes of the subacute DVT group in the present study [5]. Chung et al. have shown that large-bore (11-F) aspiration thrombectomy combined with Trerotola device is effective for DVTs older than ten days [6]. A recent study of 27 subacute DVT treated with the Angiojet device reported an anatomic success rate of 92.6% [7].

In this study, recurrences were more common in the subacute DVT group than in the acute DVT group. Symptom duration remained as an independent risk factor in multivariate analysis. This result might be because subacute DVT group suffered more severe impairments in venous function than acute DVT group. When blood clots become more organized over time, they can cause more damage to vessels and valves. In addition, it may be necessary to perform more intensive manipulations to remove organized thrombi, which can result in significant mechanical damage. Results of this study suggest that timely thrombectomy should be performed to lower the risk of recurrence. In cases of subacute DVT, extended anticoagulant therapy might be required even if anatomic success has been achieved.

The results of this research indicated that early successful thrombectomy could reduce the risk of DVT recurrence. Nonetheless, this study also has limitations due to its retrospective design. The follow-up period was relatively short, and the follow-up protocol was not standardized between centers. Further long-term study is required to validate our results. Post-thrombotic syndrome and quality of life could not be analyzed based on a scoring system.

In conclusion, single-session PMT is safe and effective for immediate symptom relief in acute and subacute DVT patients. However, recurrence occurred more frequently in patients with subacute DVT than in those with acute DVT. Anatomic success of the procedure and duration of symptoms were independent predictors of DVT recurrence.
